# Posterior Reversible Encephalopathy Syndrome in a Patient With Microscopic Polyangiitis: A Case Report and Literature Review

**DOI:** 10.3389/fmed.2021.792744

**Published:** 2021-12-24

**Authors:** Jing Xu, Ying Ding, Zhen Qu, Feng Yu

**Affiliations:** ^1^Renal Division, Department of Medicine, Peking University International Hospital, Beijing, China; ^2^Renal Division, Department of Medicine, Peking University First Hospital, Institute of Nephrology, Peking University, Beijing, China; ^3^Key Laboratory of Renal Disease, Ministry of Health of China, Beijing, China; ^4^Key Laboratory of Chronic Kidney Disease Prevention and Treatment, Ministry of Education of China, Beijing, China

**Keywords:** microscopic polyangiitis, central nervous system, intracerebral hemorrhage, posterior reversible encephalopathy syndrome, case report

## Abstract

Central nervous system (CNS) is rarely involved in microscopic polyangiitis (MPA). Here, we report a 14-year-old girl with MPA who developed new-onset seizures with deterioration of renal function. Her brain CT scan and MRI showed concurrent complications of intracerebral hemorrhage and posterior reversible encephalopathy syndrome (PRES). She got remission with combinations of methylprednisolone pulse, plasma exchange, regular hemodialysis, antiseizure and antihypertension medications. Furthermore, it is crucial to exclude the adverse effect of medications such as corticosteroid and biological therapy. We searched the literatures, retrieved 6 cases of MPA with PRES and summarized their clinical characteristics.

## Introduction

Microscopic polyangiitis (MPA) is one classical type of antineutrophil cytoplasmic antibody (ANCA)-associated systemic vasculitis which mainly affects small vessels like arterioles, capillaries or venules. Unlike peripheral vasculitic neuropathy common in patients with ANCA-associated vasculitis (1), central nervous system (CNS) involvement was infrequent in MPA (2). Even so, it may lead to complications like ischemic infarction (3–5), intracerebral hemorrhage (6, 7), subarachnoid hemorrhage (8), ventricular hemorrhage (9), as well as posterior reversible encephalopathy syndrome (PRES) (10–15).

At the same time, treatment with high dose of corticosteroids and biological agents like rituximab, could also lead to CNS complications including PRES (16–19).

Herein, we reported a rare case of MPA who developed concurrent intracerebral hemorrhage and PRES.

## Case Presentation

**Figure 1:**
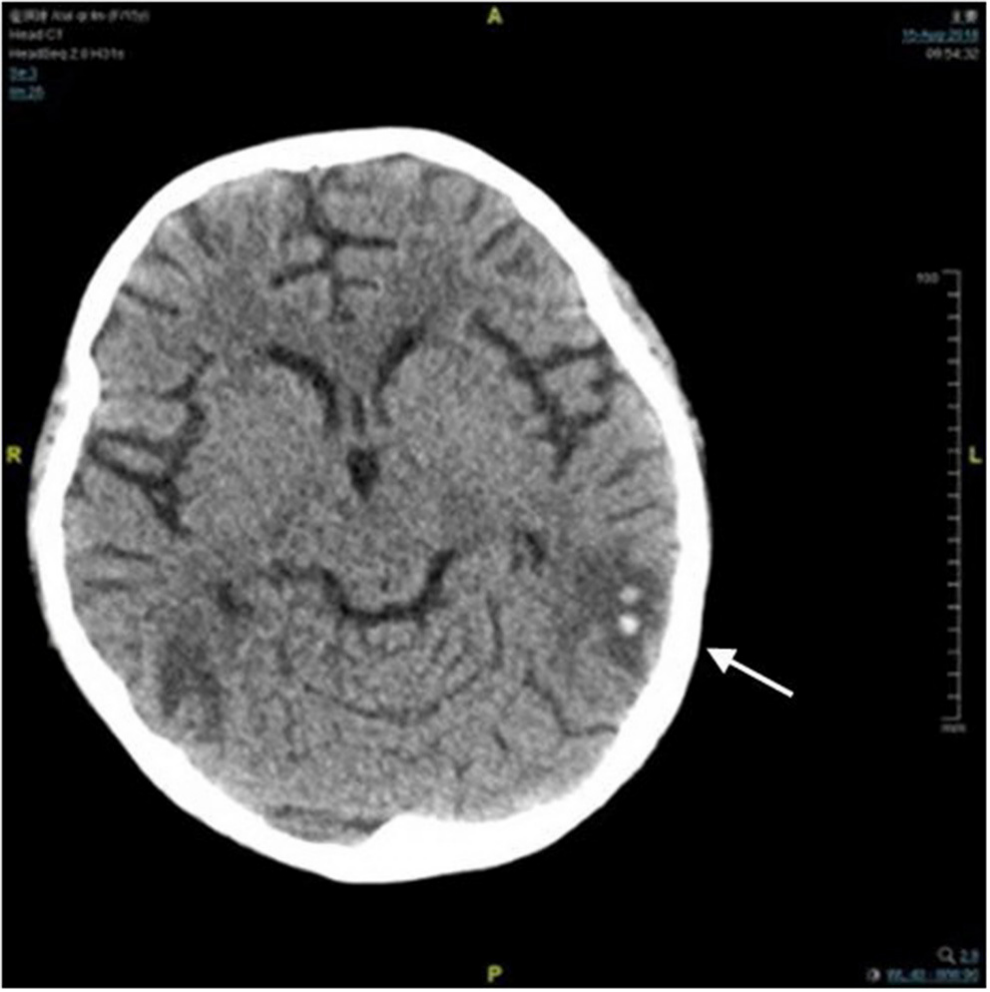
Computed tomography image of brain. There was a small amount of hemorrhage in the left temporal lobe (arrow).

**Figure 2:**
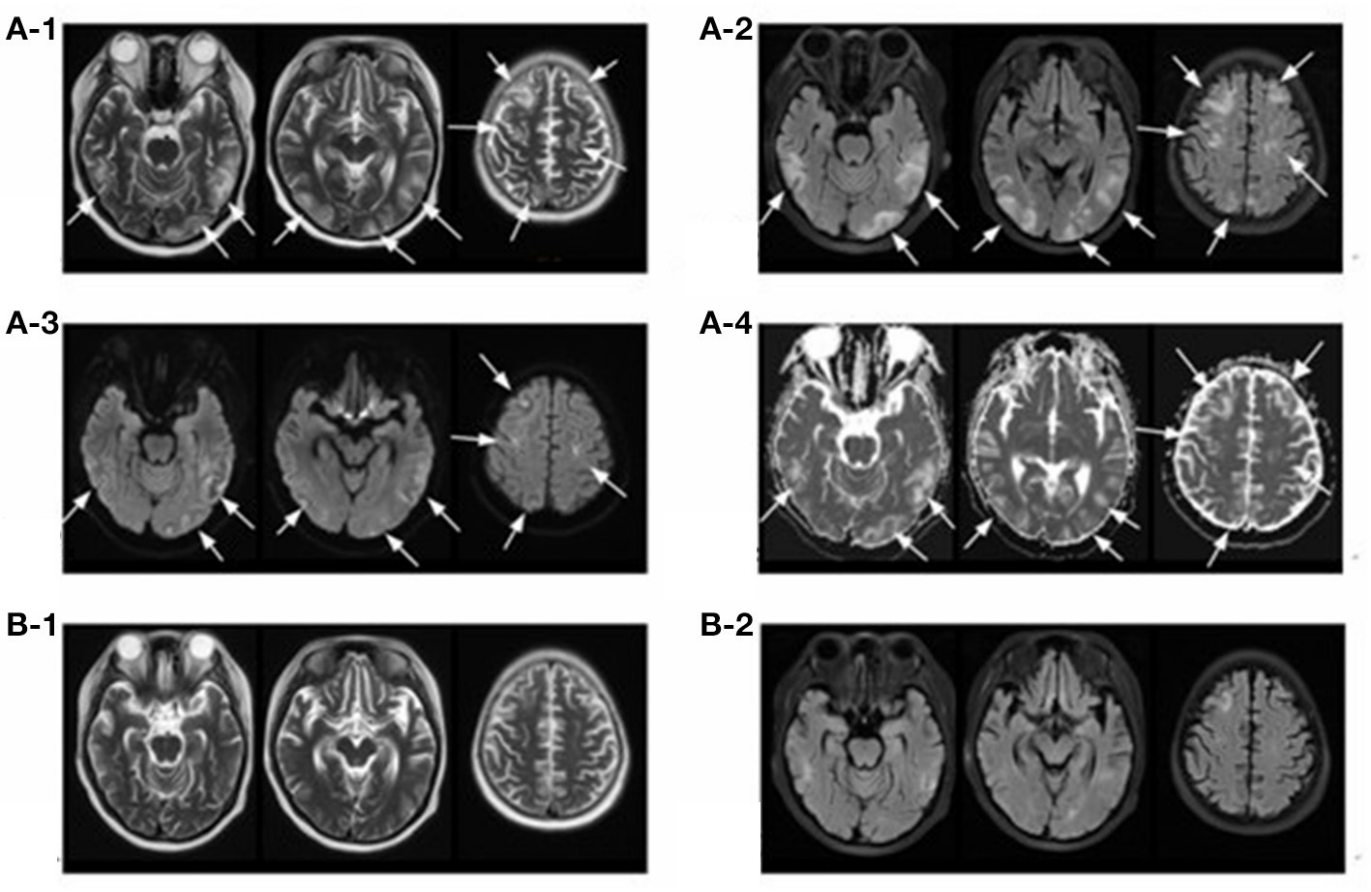
Magnetic resonance imaging (MRI) of brain. T2-weighted **(A-1)** and FLAIR **(A-2)** sequences depict hyper-intense lesions involving the bilateral cerebral hemispheres (occipital lobe, parietal lobe, temporal lobe and frontal lobe) and cerebral cortex and subcortex (arrows), most of which disappeared 17 days later **(B-1,B-2)**. Diffusion-weighted magnetic resonance imaging **(A-3)** and apparent diffusion coefficient **(A-4)** sequences of the involved regions showed isointense or hyperintensity lesions (arrows).

**Figure 3:**
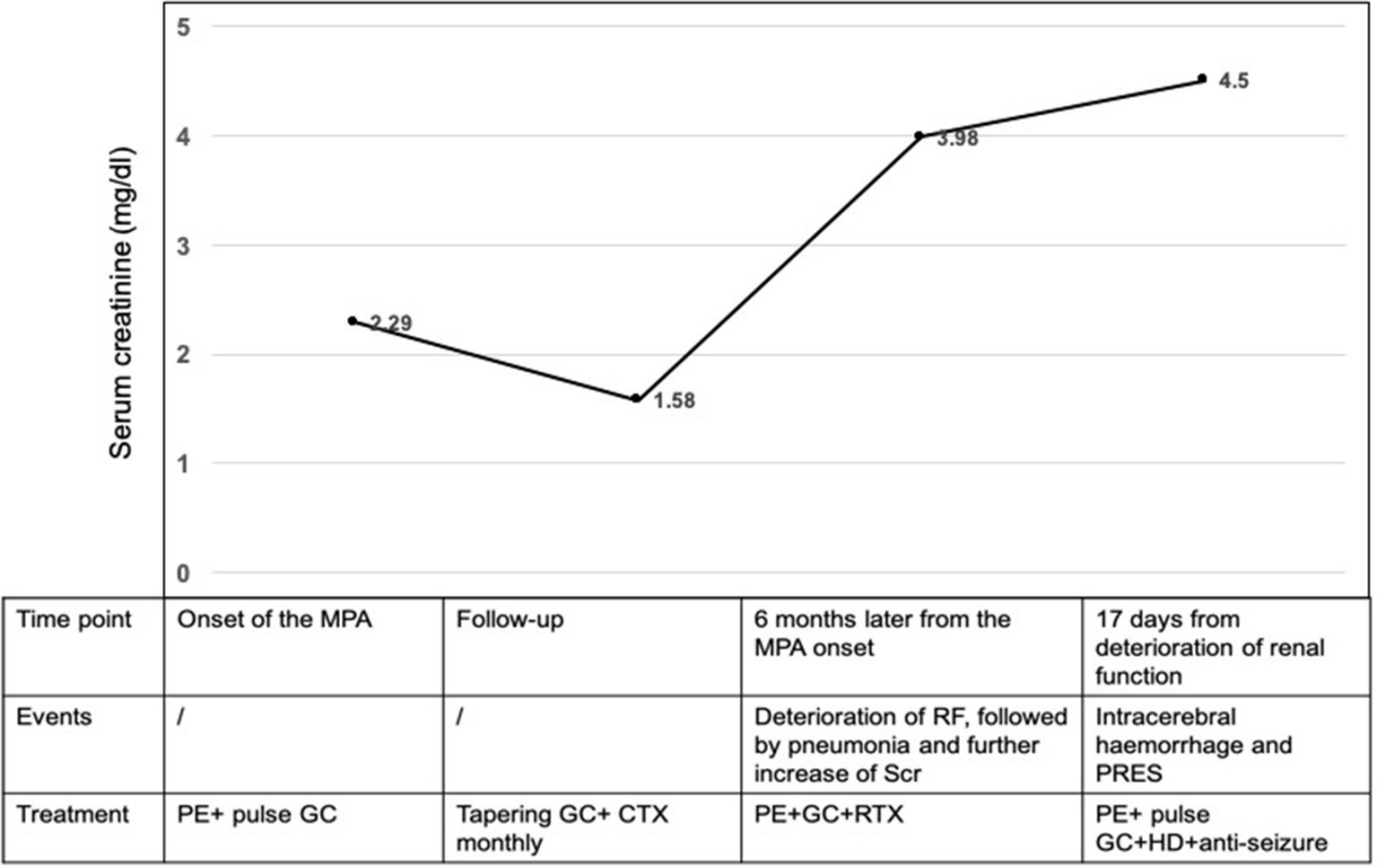
Clinical findings timeline. MPA, microscopic polyangiitis; GC, glucocorticoid; CTX, cyclophosphamide; PE, plasmapheresis; RTX, rituximab; RF, renal function; PRES, posterior reversible encephalopathy syndrome; HD, hemodialysis.

A 14-year-old girl with no significant past medical history was admitted to the local hospital in January 2018 due to lower extremities edema. Her urine examination showed proteinuria (4.95 g/24 h) and microscopic hematuria, with serum creatinine value elevated to 2.29 mg/dl. She had positive result of perinuclear antineutrophilic cytoplasmic antibody (p-ANCA) (1:32) and the myeloperoxidase-antineutrophil cytoplasmic antibody (MPO-ANCA) was 81.7 RU/ml (Enzyme linked immunosorbent assay, normal range <10 RU/ml). Her anti-glomerular basement membrane (GBM) antibody was negative. Renal biopsy was performed and reported necrotizing crescentic glomerulonephritis with pauci-immune deposits. Imaging examinations, including computed tomography (CT) scans of head and chest, showed no abnormality. She was diagnosed as MPA and was then treated with three courses of plasma exchange, methylprednisolone pulse therapy (15 mg/kg/day for 3 days), followed by oral prednisone 1 mg/kg/day (tapered gradually after 6 weeks) and intravenous cyclophosphamide (500 mg/1.73 m^2^, once a month). Her serum creatinine value gradually decreased to 1.58 mg/dl and anti-MPO antibody turned to negative. She presented with a relapse six months later with her serum creatinine value increased to 3.98 mg/dl. She was admitted to our hospital on June 27, 2018. Physical examinations showed that blood pressure was 120/80 mmHg and there were no other remarkable abnormal findings on admission. She maintained with oral prednisone of 60 mg, received another 10 rounds of plasma exchange and two doses of rituximab (100 and 500 mg respectively, with an interval of one week). Three days after the second infusion of 500 mg rituximab, she was complicated with bacterial pneumonia and a further deterioration of renal function (serum creatinine level increased to 4.69 mg/dl. Ceftriaxone (1 g/day) and meropenem (0.5 g/day) were then used. Seventeen days later, she suddenly developed disorder of thought and auditory hallucination followed 10 min later by tonic-clonic seizures. Her blood pressure was 150/100 mmHg and the neurological examinations had no abnormality at that time. Her head CT scan showed a small amount of hemorrhage in the left temporal lobe (Figure 1). Brain magnetic resonance image (MRI) revealed multiple foci lesions under bilateral cortex of the superior frontal sulcus, parieto-occipital and watershed area was dominant, which suggested typical PRES features (Figures 2A1–4). The brain magnetic resonance angiography (MRA) was normal. The clinical findings timeline was presented in Figure 3.

### Diagnostic Assessment and Therapeutic Interventions

In our case, the head CT was normal before admission and she didn't have any other risk factors for cerebrovascular disease. Her new-onset of consciousness changing and seizures, accompanied by brain MRI showing vasogenic edema in bilateral cerebral hemispheres, indicating the classic characteristics of PRES. Based on the progressive advanced renal failure with severe microscopic hematuria, elevated erythrocyte sedimentation rate (ESR) and C-reactive protein level, her Birmingham Vasculitis Activity Score (BVAS) at the time of PRES was 21, which indicated high disease activity. Furthermore, receiving high dose corticosteroid, and cytotoxic agents such as rituximab, might contribute to hypervolemia and vasospasm, which promoted the development of PRES (19–22). But the interval between the rituximab regimen against MPA and the onset of PRES was more than two weeks, much longer than the interval reported by previous literature varying between 6 h to 8 days (19–22), which excluded its contribution. The pneumonia and blood pressure were under well-control and her hemodialysis was regular and efficient, which did not support the participation of infection and hypertension (23, 24). Thus, PRES in our patient might mainly attributed to MPA. As the intracerebral hemorrhage could happen in 15% of patients with PRES (17), we proposed that the PRES contributed to the intracranial hemorrhage. Therefore, treatment was aimed at MPA with administration of 200 mg methylprednisolone and initiation of another seven courses of plasma exchange, in combination with regular hemodialysis (three times per week), anti-seizure medication and well blood pressure control. The patient's mental symptoms resolved within 24 h.

### Follow-Up and Outcomes

The follow-up brain MRI after 17 days showed that the degree of gyrus swelling was reduced, most of the abnormal high signal shadows disappeared and multiple small ischemic lesions were found under bilateral frontal-parietal cortex (Figures 2B1,2). Unfortunately, her renal function did not recover with maintenance hemodialysis and oral prednisone gradually tapered after 6 weeks. Her peripheral-blood CD19+ B-cell counts decreased to 1/μl after two times of rituximab infusion and increased to 5/μl six months later when she received another time of rituximab infusion (100 mg) as part of maintenance therapy. She was content with her treatment since the disease was controlled and stabilized. No PRES relapsed and she received a deceased donor kidney transplantation in December 2019. She observed the strict and routine follow-up with treatments to prevent graft rejection, and her renal function and central nervous system condition kept stable until now.

## Discussion

We herein report a teenage girl with active MPA and advanced renal injury who was treated with corticosteroid and rituximab, then developed PRES with intracerebral hemorrhage presenting with consciousness disturbance and seizure. In most MPA cases, it was characterized by rapidly progressive glomerulonephritis, lung hemorrhage and interstitial pneumonitis, while the CNS involvement was reported less frequently (18).

Besides MPA, there were some other predisposed factors related to PRES should be distinguished in our patient: (1) The adverse effect of rituximab: The time interval between the infusion of rituximab and PRES in our patients was much longer than reported in previous literature which has been stated in the previous paragraph (19–22). Besides, there was no PRES recurrence during the re-administration of rituximab to the patient which helped to rule out the participation of RTX (19–22). (2) Renal failure: Renal failure could occur in up to 55–57% of the PRES cases reported in the literatures (25), in which the uremic toxins accumulation could lead to endothelial dysfunction and account for the development of PRES. Thus, renal failure may act as the “second-hit” in our case. Taken together, we proposed that PRES might be mainly attributed to MPA with participation of its associated renal dysfunction in the patient.

Furthermore, although the differentiation of which therapy actually helped the case was difficult, it was noticed that our treatment project to the patient was mainly targeted on MPA *per se*. The PRES was alleviated and did not relapse with the MPA stabilization which also supported our previous hypothesis that the patient's PRES might be the neurologic involvement of ANCA associated vasculitis.

Although not fully understood, endothelial dysfunction and abnormal cerebral blood flow were key factors in the pathophysiological changes underlying PRES (16, 24–26). MPA could induce inflammation within the vasculature of the CNS and result in ischemic or hemorrhagic damage to the brain parenchyma with resultant focal or generalized neurologic deficits (3–9). In PubMed, only 6 cases of MPA with PRES were reported (10–15) (More details in Table 1). In conclusion, five of them were females and one was male, with a mean age of 43.8 years (range 10–76 years). The duration from MPA onset to PRES varied from 2 days to 3 years. Two cases had hypertension at baseline and renal failure was found in all patients (serum creatinine value ranging from 1.2 to 24.48 mg/dL). All cases received immunosuppressive therapy such as high-dose steroids, plasma exchanges, etc. They responded well to the supportive therapy combining with hemodialysis, anti-seizure medication and antihypertensive drugs. During the follow-up, two cases presented with PRES recurrence, which might be attributed to quickly tapering of steroids, irregular hemodialysis, or relapse of vasculitis.

**Table 1:** Cases describing microscopic polyangiitis with posterior reversible encephalopathy syndrome.

**No**	**References**	**Case presentation**	**Imaging of the brain**
1	Tajima et al. (10)	A 76-year-old woman who was diagnosed as isolated oculomotor neuropathy associated with MPA and the administration of oral prednisolone was started. Seven days later however, she suddenly began to complain of a headache and her consciousness was disturbed. Her blood pressure was 136/86 mm Hg. PRES associated with p-ANCA positive vasculitis was suspected and one gram of methylprednisolone was administered. Though her consciousness returned to normal, the patient developed pulmonary hemorrhage which did not respond to any medication. She eventually died of multiple organ failure. Her renal function estimated by creatinine was 0.8–1.2 mg/dl and was well-controlled until the end of her life.	Brain MRI examinations revealed high FLAIR and T2 signal intensities in the bilateral parietal, occipital and right frontal lobes. There were no significant signal alterations in the DWI and no apparent changes in the ADC map. With further steroid treatment, after three weeks, the previously observed high FLAIR and T2 signal intensity lesions had disappeared.
2	Wacker et al. (11)	A 51-year-old woman with pulmonary–renal syndrome was diagnosed as MPA with renal failure (creatinine 3.2 mg/dl). Despite the addition of several antihypertensive drugs, her blood pressure was rising up to 180/110 mm Hg. Twenty days after immunosuppressive treatment was started, the patient suffered from severe headache, a generalized seizure and complete visual loss. Methylprednisolone pulse therapy was commenced and anti-convulsant therapy started. Over the next days, seizures subsided and the patient completely regained vision. She remained in complete remission thereafter.	MRI images showed symmetrical subcortical edema. Diffusion-weighted sequences suggested cerebral ischemia. MRA showed severe bilateral narrowing of M1 and M2 cerebrovascular segments. Repeated cerebral MRA demonstrated full resolution of previously narrowed vessels and subcortical bilateral lesions within 6 weeks.
3	Fuentes et al. (12)	A 72-year-old female presented with chest discomfort, lower extremity edema, and fatigue for 2 days. The initial physical exam revealed a blood pressure at 139/80 mm Hg. A strongly positive MPO-ANCA together with the presence of crescentic glomerulonephritis on kidney biopsy were consistent with the diagnosis of MPA. The patient was then on immunosuppressive treatment with high-dose steroids. She suddenly developed severe headache followed hours later by tonic-clonic seizures. Her blood pressure at that time was 153/101 mm Hg. She responded well to the combination of hemodialysis, antiseizure medication, and blood pressure control. Immunosuppressive therapy with high-dose steroids, was continued throughout this episode. She regained consciousness and was in her baseline neurologic state in ~1 week.	Brain MRI examinations revealed high Flair and T2 signal intensities in the bilateral occipital lobes.
4	Patel et al. (13)	A 40-year-old man developed sudden blackout in front of his eyes followed by involuntary left-facial twitching and a brief episode of unresponsiveness. He had such three episodes in 1 day and was found to have a blood pressure of 160/90 mm Hg on admission. Renal failure was revealed by raised serum creatinine (24.48 mg/dl) with a positive p-ANCA and pauci-immune necrotizing crescentic glomerulonephritis. MRI of the brain revealed features of PRES. He was given intravenous pulse methylprednisolone for 3 days and recovered quickly. Two months later, the patient presented with recurrent seizures with repeat MRI of the brain revealing features of PRES. The reason behind the event was related to two missed sessions of hemodialysis and the steroid dose being tapered. Symptoms resolved with intensive hemodialysis sessions and up-titration in the dose of steroids. He underwent renal allograft transplantation, after which he showed good clinical recovery.	MRI of the brain revealed areas of altered signal intensity in right-posterior temporal, bilateral medial basal ganglionic area, inferior and parasagittal bilateral occipital regions in the subcortical region showing hyper-intensity in T2-weighted and inversion recovery images, isointense on T1-weighted and no restricted diffusion, suggestive of PRES. MRA of the brain was normal.
5	Wang et al. (14)	A 10-year-old girl with pauci-immune glomerulonephritis, and a positive p-ANCA was diagnosis with MPA. She had worsening renal function with creatinine increasing to 6.3 mg/dl. She was treated with pulse methylprednisolone, intravenous cyclophosphamide and plasmapheresis. Four days later, she developed new-onset seizure activity. Her blood pressure, which was previously in the reference range at 128/82 mm Hg, was elevated at 170/100 mm Hg. MRI of the brain revealed findings consistent with PRES. she was started on increased dose of lisinopril and amlodipine for hypertension and ziprasidone for agitation and hallucinations. She recovered within 2 days. Two weeks after discharge, she presented with recurrence of generalized seizures. Repeat MRI of the brain showed recurrence of PRES. Rituximab (400 mg/m^2^ weekly for 4 weeks) was administered for her underlying vasculitis. Over the ensuing months, her condition had remained stable.	MRI of the brain showed an area of FLAIR hyper-intensity involving the cortex and subcortical white matter in left superior parietal parasagittal gyrus, consistent with PRES. Repeat MRI showed multiple hyper-intensities involving frontal, parietal, and temporo-occipital regions, more severe on the right, consistent with recurrence of PRES.
6	Bhadu et al. (15)	A 14-year-old Indian female child was diagnosed as MPO-ANCA associated vasculitis (MPA) based on clinical, immunological, histological and radiological findings. Cyclophosphamide pulse was initiated and oral prednisolone was administered. She presented 12 days later with an episode of seizure not associated with any focal neurological deficit. MRI of the brain revealed findings consistent with PRES. She was managed with antiepileptic medication, pulse methylprednisolone and cyclophosphamide. She responded well to the treatments and recovered completely.	FLAIR and T2-weighted sequences of MRI (Brain) depicted hyper-intense lesions involving the frontoparietal cortices, which showed complete resolution at 6 months.

*MPA, microscopic polyangiitis; PRES, posterior reversible encephalopathy syndrome; p-ANCA, perinuclear antineutrophilic cytoplasmic antibody; MRI, magnetic resonance image; FLAIR, fluid attenuated inversion recovery; DWI, diffusion-weighted image; ADC, apparent diffusion coefficient; MRA, magnetic resonance image; MPO-ANCA, myeloperoxidase-antineutrophil cytoplasmic antibody*.

The limitation of our case was that it was a pure clinical report and the causal relationship between MPA and PRES accompanied by intracerebral hemorrhage was not fully elucidated. Further laboratory studies like *in vitro* experiments or animal models, are needed.

In summary, this was a case which presented a MPA patient complicated with PRES. Through careful differential diagnosis. We excluded rituximab as the cause of PRES and the patient was well followed-up.

## Data Availability Statement

The original contributions presented in the study are included in the article/supplementary material, further inquiries can be directed to the corresponding author/s.

## Ethics Statement

Peking University International Hospital, Beijing, China, does not require ethical approval for reporting individual cases. Written informed consent for patient information and images to be published was provided by the patient's legally authorized representative.

## Author Contributions

ZQ and FY: conceptualization, supervision, and writing—review and editing. JX, YD, ZQ, and FY: data curation and investigation. JX and YD: writing—original draft. All authors contributed to the article and approved the submitted version.

## Conflict of Interest

The authors declare that the research was conducted in the absence of any commercial or financial relationships that could be construed as a potential conflict of interest.

## Publisher's Note

All claims expressed in this article are solely those of the authors and do not necessarily represent those of their affiliated organizations, or those of the publisher, the editors and the reviewers. Any product that may be evaluated in this article, or claim that may be made by its manufacturer, is not guaranteed or endorsed by the publisher.
